# Outcomes of Preterm Infants With Congenital Heart Defects After Early Surgery: Defining Risk Factors at Different Time Points During Hospitalization

**DOI:** 10.3389/fped.2020.616659

**Published:** 2021-01-28

**Authors:** Po-Yin Cheung, Morteza Hajihosseini, Irina A. Dinu, Heather Switzer, Ari R. Joffe, Gwen Y. Bond, Charlene M. T. Robertson

**Affiliations:** ^1^Department of Pediatrics, University of Alberta, Edmonton, AB, Canada; ^2^NICU, Northern Alberta Neonatal Program of Alberta Health Services, Edmonton, AB, Canada; ^3^School of Public Health, University of Alberta, Edmonton, AB, Canada; ^4^Wascana Children's Program, Regina, SK, Canada; ^5^PICU Stollery Children's Hospital, Edmonton, AB, Canada; ^6^Complex Pediatric Therapies Developmental Assessment Clinic at the Glenrose Rehabilitation Hospital of Alberta Health Services, Edmonton, AB, Canada

**Keywords:** prediction model, neurodevelopmental outcome, mortality, prematurity, cardiac surgery

## Abstract

**Background:** Compared with those born at term gestation, infants with complex congenital heart defects (CCHD) who were delivered before 37 weeks gestational age and received neonatal open-heart surgery (OHS) have poorer neurodevelopmental outcomes in early childhood. We aimed to describe the growth, disability, functional, and neurodevelopmental outcomes in early childhood of preterm infants with CCHD after neonatal OHS. Prediction models were evaluated at various timepoints during hospitalization which could be useful in the management of these infants.

**Study Design:** We studied all preterm infants with CCHD who received OHS within 6 weeks of corrected age between 1996 and 2016. The Western Canadian Complex Pediatric Therapies Follow-up Program completed multidisciplinary comprehensive neurodevelopmental assessments at 2-year corrected age at the referral-site follow-up clinics. We collected demographic and acute-care clinical data, standardized age-appropriate outcome measures including physical growth with calculated *z-*scores; disabilities including cerebral palsy, visual impairment, permanent hearing loss; adaptive function (Adaptive Behavior Assessment System-II); and cognitive, language, and motor skills (Bayley Scales of Infant and Toddler Development-III). Multiple variable logistic or linear regressions determined predictors displayed as Odds Ratio (OR) or Effect Size (ES) with 95% confidence intervals.

**Results:** Of 115 preterm infants (34 ± 2 weeks gestation, 2,339 ± 637 g, 64% males) with CCHD and OHS, there were 11(10%) deaths before first discharge and 21(18%) deaths by 2-years. Seven (6%) neonates had cerebral injuries, 7 had necrotizing enterocolitis; none had retinopathy of prematurity. Among 94 survivors, 9% had cerebral palsy and 6% had permanent hearing loss, with worse outcomes in those with syndromic diagnoses. Significant predictors of mortality included birth weight *z-*score [OR 0.28(0.11,0.72), *P* = 0.008], single-ventricle anatomy [OR 5.92(1.31,26.80), *P* = 0.021], post-operative ventilation days [OR 1.06(1.02,1.09), *P* = 0.007], and cardiopulmonary resuscitation [OR 11.58 (1.97,68.24), *P* = 0.007]; for adverse functional outcome in those without syndromic diagnoses, birth weight 2,000–2,499 g [ES −11.60(−18.67, −4.53), *P* = 0.002], post-conceptual age [ES −0.11(−0.22,0.00), *P* = 0.044], post-operative lowest pH [ES 6.75(1.25,12.25), *P* = 0.017], and sepsis [ES −9.70(−17.74, −1.66), *P* = 0.050].

**Conclusions:** Our findings suggest preterm neonates with CCHD and early OHS had significant mortality and morbidity at 2-years and were at risk for cerebral palsy and adverse neurodevelopment. This information may be important for management, parental counseling and the decision-making process.

## Introduction

Infants with complex congenital heart defects (CCHD) who have open-heart surgery (OHS) during the neonatal period are at risk for mortality and neurodevelopmental morbidity (1, 2). Advancements in obstetric and neonatal care have improved the overall outcomes of preterm infants (born at <37 [+0] weeks gestation) significantly with high survival rates without neurodevelopmental impairment in early childhood (3). However, when compared with term neonates, preterm infants generally have more adverse short- and long-term neurodevelopmental outcomes depending on the degree of prematurity (3, 4). It has been shown that delivery before 39–40 weeks of gestational age (GA) is associated with higher mortality and worse neurodevelopmental outcomes for those infants with CCHD having cardiac surgery (5–12).

Despite the adverse outcome compared with that of term infants, there is little information available regarding “gestation-related details” and predictors of outcomes in early childhood of preterm neonates with CCHD and early OHS at “different time points during hospitalization.” This information is important for management of the infants, parental counseling and informing the decision-making process. We therefore aimed to describe the growth, disability, functional, and neurodevelopmental outcomes in early childhood of preterm infants with CCHD who had OHS with cardiopulmonary bypass (CPB) by 6 weeks corrected age. The secondary aims of the study included the identification of risk factors for mortality and adverse functional outcomes at each of five time points that we believe are important moments of clinical decision-making and family counseling: (i) before and at birth, (ii) pre-operative, (iii) day 1 post-operatively, (iv) post-operatively day 5, and (v) at first hospital discharge.

## Methods

We studied all preterm infants with CCHD who received OHS with CPB at 6 weeks of corrected age or less at the Stollery Children's Hospital in Edmonton, Alberta, Canada, between 1996 and 2016. Demographic, clinical and outcomes data were obtained from the Western Canadian Complex Pediatric Therapies Follow-up Program. This program maintains a prospectively collected registry and database including demographic, acute-care clinical and long-term neurodevelopmental outcomes in all infants who have complex cardiac surgery (CCS) at 6 weeks of age or less (13). The clinical information includes pre-operative, intra-operative, and post-operative data of these infants during the first hospital stay when the OHS was performed. Details of the methodology of this program have been previously published (13, 14). All neonates had standard genetic testing and geneticist assessment as appropriate. As per neuroimaging study protocol in these preterm infants, all had pre-operative cranial ultrasound examination with magnetic resonance imaging as indicated by clinical and neurologic findings. The occurrence of necrotizing enterocolitis and retinopathy of prematurity for this project was obtained by retrospective chart review. Consents were obtained from the patient's legal guardian. The study was approved by institutional ethics boards at all six follow-up sites.

### Outcomes

Disability and developmental assessments at a corrected age of 2 years were completed by multidisciplinary teams at the follow-up referral sites, of Winnipeg, Manitoba; Regina and Saskatoon, Saskatchewan; Calgary, Alberta; Vancouver, British Columbia, and the Glenrose Rehabilitation Hospital, Edmonton, Alberta, Canada. Standardized age-appropriate outcome measures included physical growth with calculated *z-*scores (15, 16), a functional measure (17) and a neurodevelopmental test of cognitive, language, and motor skills (18). Pediatricians experienced in developmental follow-up examined each child for evidence of cerebral palsy (defined as a group of permanent disorders of the development of movement and posture, causing activity limitation, that are attributed to non-progressive disturbances that occurred in the developing fetal or infant brain) (19) or visual impairment (defined as corrected visual acuity in the better eye of <20/60). Permanent hearing impairment was defined as responses in the better ear of >40 dB at any frequency from 250 to 4,000 Hz (13).

A parent completed questionnaire, the Adaptive Behavior Assessment System, second edition (ABAS-II) determined child functional abilities (17). The ABAS-II measures realistic, independent behaviors of young children and the effectiveness of interactions with others, while considering the community context. Thus, this rating scale measures daily living skills, that is, what children can do, without the assistance of others. The ABAS-II for children under age 6 years consists of nine skill areas grouped into three composite domains: Conceptual—communication, functional pre-academics, and self-direction; Practical—home living, health and safety, community use and self-care; and Social—leisure and social. A separate rating for motor skills is added to the nine skill areas to determine the overall score, the General Adaptive Composite (GAC) score. Age-based United States population norms for children have mean of 100 and a standard deviation (SD) of 15; skill areas have a mean (SD) of 10(3) (17). The ABAS-II GAC was used as the main outcome measure for analysis as it was available for all children.

Certified pediatric psychologists and psychometrists administered the Bayley Scales of Infant and Toddler Development, third edition (Bayley-III) (18). The Bayley-III is a technically sound instrument of early childhood development, with strong internal consistency, high test–retest stability and employs a representative sample based on United States demographics. This study corrected for age up to 2 years as recommended (18). The Cognitive Scale is made up of largely non-verbal activities involving memory, problem solving, and manipulation of the physical world. The Language Scale is composed of Receptive and Expressive Communication subtests. The Motor Scale includes Fine Motor (visual–motor integration, visual–spatial, and fine-motor control skills), and the Gross Motor (large body movements, mobility, and complex movements) subtests. The population mean(SD) is 100(15) (18). As our study spans over 2 decades and the Bayley-III was published in 2006, only 71 of the 94 survivors have these results and hence this measure was not used as the main outcome measure.

Anonymous data were returned to the central site for adjudication by one of the investigators (13). Maternal education was indicated by years of schooling. The Blishen Index, population mean (SD) of 43 (13) and based on employment of the main wage earner of each family, was used to reflect socioeconomic status (20).

## Statistical Analysis

CCHD were coded according to cardiac anatomy, particularly the presence of single ventricle shown to be predictive of perioperative mortality (1), and perioperative physiology as described previously (13). Mortality and outcomes were analyzed as categorical or continuous variables.

***Objective 1***: First, we conducted a descriptive analysis to explore the distribution of mortality and each neurodevelopmental outcome using mean, SD, median, and interquartile range (IQR). Student *t*, Fisher's Exact and Chi-square tests were used to compare groups.

***Objective 2***: To determine potential predictors of adverse outcomes [mortality, and ABAS-II-GAC scores], we used univariate and multiple variable regression analyses.

a. *Univariate analyses*: Using logistic regression, we compared the demographic and clinical variables at each time point between the surviving and non-surviving groups of infants during the first hospital stay for OHS. Using linear regression, we analyzed predictors of the ABAS-II GAC scores as a continuous variable. Following these comparisons, risk factors with *P* < 0.1 were selected for the multiple variable regression models.

b. *Multiple variable analyses*: The effect of risk factors for mortality and ABAS-II GAC scores was further examined in multiple variable models using five clinical time points for analyses. We chose these five periods as they are important time points informing clinical management decisions and family counseling. The five time points were: (i) before and at birth, (ii) pre-operative, (iii) post-operative day 1, (iv) post-operative day 5, and (v) before hospital discharge. Predictive analyses were performed at each time point with the retainment of risk factors which had *P* < 0.1 in the previous clinical period. Because of the large association of chromosomal abnormalities with adverse outcome, the prediction of the ABAS-II GAC scores was further completed with the non-syndromic 76 children. Only potential predictor variables that occurred in 10% of more of the total population were entered in the regressions. In view of the small number of infants in some GAs, we also analyzed the effects of GA on outcomes using three different categorical bands (28–31, 32–34, and 35–37 weeks).

Continuous variables were tested for the normality of their distribution and are presented as means (SD) or medians (IQR). Odds Ratio (OR) and Effect Size (ES) are reported with 95% confidence intervals [CI]. Significance was determined as *P* ≤ 0.05. Data analyses were performed using *R* software V3.6.1 (21).

## Results

### General Description

From September 1996 to December 2016, all 115 preterm infants with CCHD who received OHS at ≤6 weeks corrected age at the Stollery Children's Hospital were registered. These 115 children made up 10.7% of all children at age 6 weeks and under having complex cardiac surgery with CPB at this institution during this time. The demographic and clinical characteristics are shown in Table 1. Of these infants, 11 (9.6%) and 21 (18.3%) died before first hospital discharge and by the age of 2 years corrected age, respectively. The reasons for death before first discharge included multiple organ dysfunction (9), brain hemorrhage (1) and septic shock (1). The causes of additional deaths included cardiac failure with chronic illness (5), thrombo-embolic event (1), probable non-accidental injury (1), sepsis (1) and unknown (2). Prior to the first surgery, 7 (6.1%) neonates had cerebral injuries known to be risk factors for disability [including infarction (3), periventricular leukomalacia (2), and grade 3–4 intraventricular hemorrhage (2)]. In addition, 7 (6.1%) preterm infants had necrotizing enterocolitis. None had retinopathy of prematurity.

**Table 1:** Clinical characteristics of 115 preterm infants with complex congenital heart defects who had open heart surgery at ≤6 weeks corrected postnatal age from September 1996 to December 2016.

**Time periods**	**All preterm infants (*n* = 115)**	**Survivors at 2-years corrected age (*n* = 94)**	**Non-survivors at 2-years corrected age (*n* = 21)**	***P*-value^**a**^**
**Before or at birth**				
Year of birth	2008.4(5.5) 2010[2004, 2013]	2008.4(5.6)	2007.9(5.3)	0.610
Gestational age, weeks	34.4(1.9) 34 [34, 36]	34.4(2.0)	34.4(1.7)	0.994
*Gestational age groups*				
< = 32 weeks	20(17%)	16(17%)	4(19%)	0.464
33-34 weeks	22(19%)	20(21%)	2(10%)	
35-36 weeks	73(63%)	58(62%)	15(71%)	
Birthweight, grams	2,339(637) 2,340 [1,960, 2,690]	2,397(638)	2,079(587)	0.038
*Birthweight groups*				
≤1,499 g	11(10%)	7(7%)	4(19%)	0.259
1,500–1,999 g	18(16%)	14(15%)	4(19%)	
2,000–2,499 g	42(37%)	34(36%)	8(38%)	
≥2,500 g	44(38%)	39(41%)	5(24%)	
Birthweight, *z*-score	−0.01(1.11) −0.08 [−0.74, 0.54]	0.12(1.1)	−0.61(0.98)	0.005
Small for gestational age	11(10%)	7(7%)	4(19%)	0.221
Head circumference, cm	31.6(2.4) 32 [30, 33.5]	31.7(2.4)	31.3(2.6)	0.489
Head circumference, *z*-score	0.29(1.1) 0.47 [−0.42, 0.92]	0.33(1.1)	0.13(1.2)	0.460
Sex, male	74(64%)	60(64%)	14(67%)	0.999
5-min Apgar score	7.7 (1.5) 8 [7, 9]	7.7 (1.5)	7.8(1.5)	0.871
Multiple birth	19(17%)	15(16%)	4(19%)	0.984
Chromosomal/syndromic diagnoses	21(18%) 9-del22q11 2-Trisomy 21 3-VACTERL 3-CHARGE 2-Turner 1-17p13.3del 1-3 duplication, optic nerve hypoplasia	18(19%) 8-del22q11 2-Trisomy 21 3-VACTERL 2-CHARGE 2-Turner 1-17p13.3del	3(14%) 1-del22q11 1-CHARGE 1-3 duplication, optic nerve hypoplasia	0.834
Cardiac defect, single ventricle	31(27%)	18(19%)	13(62%)	<0.001
**Pre-operative**				
Antenatal diagnosis	62(54%)	49(52%)	13(62%)	0.568
Highest plasma lactate, mmol/L	3.5(3.2) 2.5 [1.5, 42]	3.3(3.4)	4.4(2.4)	0.07
Lowest arterial pH	7.27(0.12) 7.31 [7.20, 7.35]	7.29(0.13)	7.22(0.09)	0.001
Lowest PaO_2_, mmHg	37.9(13.2) 38 [30, 44]	39.6(13.4)	30.6(9.5)	0.001
Lowest base deficit, mmol/L	−4.6(4.9) −4 [−7, −1]	−4.1(5.2)	−6.7(2.9)	0.003
Highest inotrope score^b^	6.5(16.8) 0 [0, 8]	4.8(14.8)	13.8(22.8)	0.097
Highest serum creatinine, umol/L	63.2(30.5) 55 [41, 85]	58.3(28.1)	84.9(32.3)	<0.001
Brain insults (by imaging) considered high risk for adverse outcome	7(6%)	6(6%)	1(5%)	0.899
Ventilation, days	8.6(14.4) 5 [0,10]	7.5(13.1)	13.4(18.8)	0.184
**Operative, post-operative day 1**				
Postnatal age, days	23.5(23.4) 14 [9, 33]	24.1(23.9)	20.8(21.4)	0.528
Post-conceptual age, days	261.8(27.7) 261 [255, 271]	261.8(29.6)	262.1(16.9)	0.945
CPB time, min	117.6(44.1) 111 [90, 142]	115.3(42.7)	128.2(49.4)	0.274
Cross clamp time, min	62.1(27.4) 56 [46, 78]	62.8(27.9)	58.9(25.4)	0.537
DHCA, used	80(70%)	60(64%)	20(95%)	0.010
Highest plasma lactate, mmol/L	6.2(3.9) 4.9 [3.5, 5.8]	5.5(3.3)	9.3(4.8)	0.002
Lowest arterial pH	7.27(0.08) 7.28 [7.22, 7.32]	7.28(0.07)	7.21(0.11)	0.009
Lowest PaO_2_, mmHg	54.1(19.6) 54 [35, 68]	57.3(18.8)	39.6(16.6)	<0.001
Lowest base deficit, mmol/L	−3.5(4.7) −3 [−5,−1]	−3.0(4.3)	−5.7(6.0)	0.065
Highest inotrope score^b^	12.5(11.5) 10 [6, 16]	11.0(8.0)	19.0(19.9)	0.087
Highest serum creatinine, umol/L	56.0(21.1) 59 [45, 89]	54.9(20.9)	61.3(21.7)	0.224
**Post-operative day 2–5**				
Highest plasma lactate, mmol/L	3.6(3.3) 2.5 [1.7, 4.5]	3.0(2.0)	6.2(5.9)	0.025
Lowest arterial pH	7.28(0.07) 7.29 [7.24, 7.33]	7.29(0.06)	7.24(0.10)	0.020
Lowest PaO_2_, mmHg	53.7(17.0) 54 [38, 64]	55.9(16.1)	43.6(18.0)	0.007
Lowest base deficit, mmol/L	−3.6(4.3) −3 [−6,−1]	−3.2(3.9)	−5.5(5.4)	0.068
Highest inotrope score^b^	12.6(15.7) 10 [4, 15]	10.2(8.1)	23.7(30.6)	0.058
Highest serum creatinine, umol/L	68.9(32.8) 59 [45, 89]	66.7(33.0)	78.8(30.5)	0.115
**Before discharge**				
Post-operative ventilation, days	16.5(24.1) 10 [6, 15]	11.3(10.1)	39.4(46.5)	0.012
Post-operative ICU, days	24.4(22.9) 18 [10, 31]	19.9(14.2)	44.4(37.2)	0.007
CPR	13(11%)	3(3%)	10(48%)	<0.001
Sepsis^c^	28(24%)	20(21%)	8(38%)	0.157
ECMO	14(12%)	5(5%)	9(43%)	<0.001
Open sternum, days	4.4(7.0) 4 [0, 6]	2.8(3.2)	11.4(13.0)	0.007

*Data are presented in mean(SD), median [IQR], n(%)*.

*CPB, cardiopulmonary bypass; DHCA, deep hypothermic circulatory arrest; ICU, intensive care unit; CPR, cardiopulmonary resuscitation; ECMO, extracorporeal membrane oxygenation*.

a *Students t test, chi-square or Fisher's Exact test*.

b *Inotrope score (22) = dopamine (ug/kg/min) + dobutamine (ug/kg/min) + 100 × epinephrine (ug/kg/min)*.

c *Definition of sepsis (23)*.

### Mortality Prediction

Following the five different time points when important parental counseling sessions may occur, Table 2 shows that among the risk factors studied at the before and at birth time point, birthweight *z-*scores and primary cardiac defect of single ventricle were associated with death and these factors remained significant when including variables from all time points. Other risk factors included the lowest arterial pH at the pre-operative time point, lowest arterial pH and highest lactate level post-operatively, and cardiopulmonary resuscitation (CPR) and days of post-operative ventilation before discharge. GA, postnatal age and the post-conceptual age of surgery were not statistically significantly different between survivors and non-survivors (Table 1).

**Table 2:** Predictive factors of death by 2-years corrected age in 115 preterm infants with complex congenital heart defects who had early open heart surgery from September 1996 to December 2016.

	**Univariate regression**	**Multiple logistic regression**				
**Time points**		**Time point 1, before and at birth**	**Time points 1–2, up to before first operation**	**Time points 1–3, up to end of day 1 post-operatively**	**Time points 1–4, up to day 5 post-operatively**	**Time points 1–5, for all time periods up to hospital discharge**
**1. Before or at birth**						
Birthweight, grams	1.00(1.00, 1.00) *P* = 0.041					
Birthweightgroups						
≤1,499 g	2.92(0.77, 11.11) *P* = 0.115					
1,500–1,999 g	0.5(0.09, 2.69) *P* = 0.412					
2,000–2,499 g	0.41(0.10, 1.88) *P* = 0.230					
≥2,500 g	0.22(0.05, 1.09) *P* = 0.057					
Birthweight, *z*-score	0.50(0.29, 0.81) *P* = 0.008	0.50(0.21, 1.04) *P* = 0.008	0.42(0.23, 0.76) *P* = 0.004	0.35(0.18, 0.69) *P* = 0.002	0.35(0.18, 0.69) *P* = 0.002	0.28(0.11, 0.72) *P* = 0.008
Cardiac defect, single ventricle	6.86(2.53, 19.80) *P* < 0.001	8.85(2.91, 30.92) *P* < 0.001	5.75(1.86, 17.71) *P* = 0.002	4.37(1.26, 15.18) *P* = 0.021	4.37(1.26, 15.18) *P* = 0.021	5.92(1.31, 26.80) *P* = 0.021
**2. Pre-operative**						
Lowest arterial pH	0.008(0.00, 0.28) *P* = 0.011		0.004(0.00, 0.39) *P* = 0.018	0.001(0.00, 0.28) *P* = 0.015	0.001(0.00, 0.28) *P* = 0.015	
Lowest PaO_2_, mmHg	0.91(0.86, 0.97) *P* = 0.002					
Lowest base deficit, mmol/L	0.90(0.82, 0.99) *P* = 0.037					
Highest inotrope score^a^	1.02(1, 1.06) *P* = 0.066					
Highest serum creatinine, umol/L	1.03(1.01, 1.05) *P* = 0.001					
**3. Operative, post-operative day 1**						
DHCA, used	11.33(1.46, 88.21) *P* = 0.020					
Highest plasma lactate, mmol/L	1.25(1.11, 1.43) *P* < 0.001			1.20 (1.01, 1.42) *P* = 0.041	1.20(1.01, 1.42) *P* = 0.041	
Lowest arterial pH	0.00(0.00, 0.021) *P* = 0.003					
Lowest PaO_2_, mmHg	0.94(0.91, 0.97) *P* = 0.001					
Lowest base deficit, mmol/L	0.90(0.81, 0.99) *P* = 0.024					
Highest inotrope score^a^	1.06(1.01, 1.12) *P* = 0.031					
**4. Post-operative day 2–5**						
Highest plasma lactate, mmol/L	1.31(1.12, 1.59) *P* = 0.002					
Lowest arterial pH	0.00(0.00, 0.02) *P* = 0.003					
Lowest PaO_2_, mmHg	0.95(0.925, 0.98) *P* = 0.004					
Lowest base deficit, mmol/L	0.88(0.78, 0.98) *P* = 0.025					
Highest inotrope score^a^	1.05(1.02, 1.10) *P* = 0.010					
**5. Before discharge**						
Post-operative ventilation, days	1.05(1.02, 1.10) *P* = 0.003					1.06(1.02, 1.09) *P* = 0.003
Post-operative ICU, days	1.05(1.02, 1.07) *P* < 0.001					
CPR	27.58(7.25, 138.04) *P* < 0.001					11.58(1.97, 68.24) *P* = 0.007
ECMO	13.35(3.97, 50.17) *P* < 0.001					
Open sternum, days	1.34(1.17, 1.57) *P* < 0.001					

*Odds ratio (OR) with 95% confidence intervals (CI) by univariate regression (variables with P < 0.10 included) and multiple logistic regression at 5 different time points*.

*CPB, cardiopulmonary bypass; DHCA, deep hypothermic circulatory arrest; ICU, intensive care unit; CPR, cardiopulmonary resuscitation; ECMO, extracorporeal membrane oxygenation; OR, odds ratio; CI, confidence interval*.

a *Inotrope score (22) = dopamine (ug/kg/min) + dobutamine (ug/kg/min) + 100 x epinephrine (ug/kg/min)*.

### Growth, Disability, and Neurodevelopmental Outcomes

All 94 (100%) surviving infants received neurodevelopmental evaluation at 2 years corrected age. The demographic and clinical characteristics of these surviving preterm infants with (*n* = 18, 19.1%) and without (*n* = 76, 80.9%) syndromic diagnoses are shown in the Table 3. The physical growth and neurodevelopmental outcomes are shown in Table 4. Although growth, functional, and neurodevelopmental parameters of survivors are within the population normative range, the means are shifted to the left of population norms. Family socioeconomic status, years of mother's schooling, as well as 2-year growth, did not differ between those with and without syndromes (Table 4). Eight (10.5%) of the 76 survivors without identified syndromic diagnoses had spastic cerebral palsy; of these, three had pre-operative brain insult with risk for motor disability. Visual impairment occurred equally in each group: in the syndromic group one child had bilateral optic nerve colobomata; in the non-syndromic group one child had cortical visual impairment. Of the five infants with permanent sensorineural hearing loss, only one had a syndromic abnormality. Those infants with syndromic diagnoses had worse functional and cognitive outcomes compared with those without syndromes (Table 4).

**Table 3:** Clinical characteristics of 94 surviving preterm infants after open heart surgery at 6 weeks or less corrected postnatal age from September 1996 to December 2016.

**Time periods**	**All surviving preterm infants (*n =* 94)**	**Survivors without chromosomal or syndromic diagnoses (*n =* 76)**	**Survivors with chromosomal or syndromic diagnoses (*n =* 18)**	**P-value^**a**^**
**Before or at birth**				
Year of birth	2008.4(5.6) 2010 [2004, 2013]	2008.7(5.5)	2007.8(6.1)	0.523
Gestational age, weeks	34.4(2.0) 35 [34, 36]	34.5(1.8)	34.2(2.8)	0.715
*Gestational age groups*				
≤32 weeks	16(17%)	13(17%)	3(17%)	0.855
33-34 weeks	20(21%)	17(22%)	3(17%)	
35-36 weeks	58(62%)	46(61%)	12(67%)	
Birthweight, grams	2,397(638) 2,370 [2,007, 2,706]	2,429(624)	2,261(696)	0.316
*Birthweight groups*				
≤1,499 g	7(7%)	5(7%)	2(11%)	0.707
1,500–1,999 g	14(15%)	12(16%)	2(11%)	
2,000–2,499 g	34(36%)	26(34%)	8(44%)	
≥2,500 g	39(41%)	33(43%)	6(33%)	
Birthweight, *z*-score	0.12(1.1) 0.06 [−0.61, 0.65]	0.18(1.1)	−0.13(0.91)	0.219
Small for gestational age	7(7%)	4(5%)	3(17%)	0.247
Head circumference, cm	31.7(2.4) 32 [30.5, 33.5]	31.7(2.2)	31.8(3.2)	0.930
Head circumference, *z*-score	0.33(1.1) 0.49 [−0.29, 0.93]	0.29(1.1)	0.52(1.1)	0.439
Sex, male	60(64%)	49(64%)	11(61%)	0.999
5-min Apgar score	7.7(1.5) 8 [7, 9]	7.6(1.5)	7.9(1.8)	0.512
Multiple birth	15(16%)	15(20%)	0(0%)	0.089
Cardiac defect, Single ventricle	18(19.1%)	17(22.4%)	1(5.6%)	0.180
**Pre-operative**				
Antenatal diagnosis	49(52%)	40(53%)	9(50%)	0.999
Highest plasma lactate, mmol/L	3.3(3.4) 2.1 [1.5, 3.3]	3.5(3.7)	2.2(1.1)	0.010
Lowest arterial pH	7.29(0.13) 7.31 [7.22, 7.37]	7.28(0.13)	7.30(0.09)	0.388
Lowest PaO_2_, mmHg	39.6(13.4) 39 [32, 45]	37.3(10.23)	49.1(20.0)	0.026
Lowest base deficit, mmol/L	−4.1(5.2) −3.7 [−7, −1]	−4.2(5.4)	−3.9(4.2)	0.825
Highest inotrope score^b^	4.8(14.8) 0 [0, 5]	5.2(16.2)	3.1(5.3)	0.346
Highest serum creatinine, umol/L	58.3(28.1) 52 [35, 78]	59.6(28.9)	52.5(23.8)	0.334
Brain insults (by imaging) considered high risk for adverse outcome	6(6%)	6(9%)	0(0%)	0.592
Ventilation, days	7.5(13.1) 5 [0, 10]	6.9(8.1)	10.3(25.1)	0.570
**Operative, post-operative day 1**				
Postnatal age, days	24.1(23.9) 14 [9, 34]	21.5(19.6)	35.2(35.8)	0.134
Post-conceptual age, days	261.8(29.6) 261 [255, 271]	260.1(31.5)	269(18.9)	0.126
CPB time, min	115.3(42.7) 109.5 [90, 138]	118.2(45.1)	102.9(28.6)	0.079
Cross clamp time, min	62.8(27.9) 56.5 [46, 77]	64.5(30.1)	55.4(13.4)	0.056
DHCA, used	60(64%)	51(67%)	9(50%)	0.278
Highest plasma lactate, mmol/L	5.5(3.3) 4.7 [3.4, 7.1]	5.5(3.4)	5.6(2.8)	0.860
Lowest arterial pH	7.28(0.07) 7.29 [7.23, 7.33]	7.27(0.07)	7.30(0.06)	0.074
Lowest PaO_2_, mmHg	57.3(18.8) 56.5 [40.8, 74.3]	56.3(18.3)	61.7(20.9)	0.324
Lowest base deficit, mmol/L	−3.0(4.3) −3 [−5, −1]	−3.2(4.6)	−2.4(2.8)	0.398
Highest inotrope score^b^	11.0(8.0) 10 [5, 15]	10.5(7.9)	13.3(8.4)	0.208
Highest serum creatinine, umol/L	54.9(20.9) 51 [40, 66]	54.5(19.8)	56.6(25.5)	0.748
**Post-operative day 2–5**				
Highest plasma lactate, mmol/L	3.0(2.0) 2.4 [1.6, 3.9]	3.0(2.1)	3.1(1.6)	0.905
Lowest arterial pH	7.29(0.06) 7.30 [7.26, 7.33]	7.29(0.06)	7.28(0.05)	0.416
Lowest PaO_2_, mmHg	55.9(16.1) 55 [47.5, 64.5]	55.7(16.3)	56.8(15.4)	0.788
Lowest base deficit, mmol/L	−3.2(3.9) −3 [−5, −1]	−3.0(3.9)	−3.8(3.7)	0.431
Highest inotrope score^b^	10.2(8.1) 9 [4, 14]	10.1(8.5)	10.6(5.9)	0.778
Highest serum creatinine, umol/L	66.7(33.0) 57 [43, 88]	65.1(30.2)	73.2(43.4)	0.460
**Before discharge**				
Post-operative ventilation, days	11.3(10.1) 9 [6, 13]	11.4(11.0)	11.0(4.7)	0.802
Post-operative ICU, days	19.9(14.2) 15 [10, 26]	18.6(13.7)	25.4(15.4)	0.070
CPR	3(3%)	3(4%)	0(0%)	0.912
Sepsis^c^	20(21.3%)	17(22.4%)	3(16.7%)	0.755
ECMO	5(5%)	4(5%)	1(6%)	0.999
Open sternum, days	2.8(3.2) 3 [0, 5]	3.0(3.2)	2.4(2.9)	0.520

*Data are presented as mean(SD), median [IQR], n(%)*.

*CPB, cardiopulmonary bypass; DHCA, deep hypothermic circulatory arrest; ICU, intensive care unit; CPR, cardiopulmonary resuscitation; ECMO, extracorporeal membrane oxygenation*.

a *Students t-test, chi-square or Fisher's Exact test*.

b *Inotrope score (22) = dopamine dose (ug/kg/min) + dobutamine (ug/kg/min) + 100 x epinephrine (ug/kg/min)*.

c *Definition of sepsis (23)*.

**Table 4:** Growth and neurodevelopmental outcomes at 2-years corrected age for 94 surviving preterm infants after early open heart surgery from September 1996 to December 2016.

	**All surviving preterm infants (*n* = 94)**	**Survivors without chromosomal or syndromic diagnoses (*n* = 76)**	**Survivors with chromosomal or syndromic diagnoses (*n* = 18)**	***P*-value^**a**^**
**Family background**				
Socio-economic index^b^	44.6(13.7) 43 [34, 54]	44.5(13.9)	44.9(13.1)	0.902
Mother's schooling, years	13.4(3.2) 13 [12, 16]	13.5(3.4)	12.9(2.1)	0.367
**Growth**				
Weight, *z*-score	−0.71(1.33) −0.66 [−1.73, 0.00]	−0.70(1.31)	−0.75(1.43)	0.867
Length, *z*-score	−0.96(1.44) −0.97 [−1.95, 0.00]	−0.88(1.43)	−1.31(1.52)	0.256
Head circumference, z-score	−0.39(1.36) −0.60 [−1.45, 0.22]	−0.41(1.37)	−0.31(1.38)	0.782
**Disability**				
Cerebral palsy (19)	8(9%)	8(11%)	0(0%)	0.346
Cerebral palsy types, all spastic		5- hemiplegia; 1-quadriplegia; 1-triplegia; 1-monoplegia	0	
Visual impairment	2(2%)	1(1%)	1(6%)	Not applicable
Hearing loss	6(6%)	4(5%)	2(11%)	0.323
Hearing loss types		3-bilateral sensorineural; 1-unilateral sensorineural	1-bilateral sensorineural; 1-permanent conductive	
**Functional scores (ABAS-II)** **(****17****)**				
General adaptive composite	83.7(18.1) 85 [70, 92]	86.2(17.8)	70.7(14.1)	0.004
Conceptual composite: -Communication -Functional pre-academics -Self-direction	88.3(17.1) 8.4(3.4) 8.3(2.9) 8.6(3.5)	89.7(17.0) 8.6(3.5) 8.4(2.9) 8.9(3.5)	76.9(12.5) 6.6(2.2) 7.4(2.9) 6.3(1.9)	0.060 0.137 0.403 0.062
Practical composite: -Home living -Health and safety -Community use -Self-care	84.9(17.5) 9.0(3.2) 8.2(3.5) 8.9(3.1) 5.4(3.2)	86.5(17.3) 9.2(3.2) 8.5(3.5) 9.2(3.0) 5.7(3.3)	72.1(14.1) 7.7(3.0) 5.7(2.4) 6.9(2.9) 3.4(2.1)	0.039 0.268 0.045 0.053 0.085
Social composite: -Leisure -Social	89.8(18.1) 8.6(3.3) 8.7(3.4)	91.1(18.2) 8.9(3.3) 8.9(3.4)	79.1(13.2) 6.4(2.4) 7.1(2.5)	0.098 0.063 0.207
Motor skill	8.3(3.9)	8.4(3.9)	7.1(3.9)	0.381
**Neurodevelopment scores (Bayley-III)** **(****18****)**, ***n*** **=** **71**				
Cognitive	87.6(15.4) 90 [80, 100]	90.2(13.6) (*n* = 54)	80.1(17.1) (*n* = 17)	0.018
Language	82.4(17.1) 83 [71, 94]	84.4(17.1) (*n* = 54)	74.9(17.3) (*n* = 17)	0.055
Motor	85.1(16.4) 85 [75, 94]	87.1(15.7) (*n* = 54)	78.7(17.4) (*n* = 17)	0.062

*Data are presented in mean(SD), median [IQR], n(%)*.

a *Students t test or Fisher's Exact test*.

b *Blishen Index (20)*.

### Functional Outcome Prediction

As there was a significant contribution of chromosomal/syndromic diagnoses to ABAS-II GAC at 2 years corrected age in the 94 surviving preterm infants [ES(95%CI) −13.8(−22.5, −5.1)], we sought possible predictors of outcome including only the 76 infants without syndromic abnormalities (Table 5). We did not evaluate the effects of pre-operative brain injury because of the small number of infants affected (*n* = 6). Of the birthweight groups, those with a weight of 2,000–2,499 g (*n* = 26) had ABAS-II GAC scores significantly below the others (*n* = 50), [76.9 ± 13.7 vs. 90.5± 16.6, *t* = 3.593, *P* = 0.001]. This birth weight of 2,000–2,499 g was associated with the ABAS-II GAC at each of the 5 different time points. The adjusted *R*^2^ of the variance in ABAS-II GAC scores at 2 years accounted for in the multiple logistic models was 0.263 before discharge from the hospital (Table 5). The explained variance was 0.137 at birth with the predictor birthweight of 2,000–2,499 g alone. Other statistically significant predictive risk factors also included post-conceptual age of surgery, lowest arterial pH on day 2–5 post-operatively, and sepsis. GA, birthweight *z-*sores and postnatal age of surgery were not found to be independent variables associated with functional outcome.

**Table 5:** Predictive factors of General Adaptive Composite (ABAS-GAC) at 2-years of corrected age for 76 preterm infants without chromosomal/syndromic abnormalities with complex congenital heart defects who had early open heart surgery from September 1996 to December 2016.

	**Univariate regression**	**Multiple linear regression**				
**Time points**		**Time point 1, before and at birth**	**Time points 1–2, up to before first operation**	**Time points 1–3, up to end of day 1 post-operatively**	**Time points 1–4, up to day 5 post-operatively**	**Time points 1–5, for all time periods up to hospital discharge**
**1. Before or at birth**						
Year of birth	0.66(−0.03, 1.36) *P* = 0.060					
Gestational age groups						
< = 32 weeks	9.70(−0.36, 19.76) *P* = 0.058					
Birthweight groups						
2,000–2,499 g	−13.61(−21.16, −6.06) *P* = 0.001	−13.61(−21.16, −6.06) *P* = 0.001, Adj *R*^2^= 0.137	−12.74(−20.16, −5.32) *P* = 0.001, Adj *R*^2^= 0.137	−12.74(−20.16, −5.32) *P* = 0.001, Adj *R*^2^= 0.137	−11.88(−18.93, −4.83) *P* = 0.001, Adj *R*^2^= 0.137	−11.60(−18.67, −4.53) *P* = 0.002, Adj *R*^2^= 0.137
Head circumference, *z*-score	2.92(−0.54, 6.38) *P* = 0.097					
**2. Pre-operative**						
Highest plasma lactate, mmol/L	−0.11(−2.11, −0.04) *P* = 0.042					
Lowest arterial pH	3.36 (0.52, 6.20) *P* = 0.021		2.86(0.19, 5.53) *P* = 0.036 Change in Adj *R*^2^ = 0.040	2.86(0.19, 5.53) *P* = 0.036 Change in Adj *R*^2^ = 0.040	3.01(0.48, 5.54) *P* = 0.021 Change in Adj *R*^2^ = 0.040	
Lowest PaO_2_, mmHg	0.32(−0.05, 0.70) *P* = 0.092					
Lowest base deficit, mmol/L	0.66(−0.05, 1.37) *P* = 0.067					
Highest Inotrope score^22^	−0.26(−0.49, −0.02) *P* = 0.057					
Highest serum creatinine, umol/L	−0.17(−0.30, −0.05) *P* = 0.009					
**3. Operative, post-operative day 1**						
Post-conceptual age	−0.11(−0.23, 0.02) *P* = 0.089				−0.11(−0.22, −0.01) *P* = 0.036 Change in Adj *R*^2^ = 0.037	−0.11(−0.22, −0.00) *P* = 0.044, Change in Adj *R*^2^ = 0.033
Highest inotrope score^a^	−0.58(−1.05, −0.10) *P* = 0.019					
**4. Post-operative day 2–5**						
Lowest arterial pH	7.18(0.98, 13.37) *P* = 0.024				7.14(1.63, 12.63) *P* = 0.018 Change in Adj *R*^2^ = 0.046	6.75 (1.25, 12.25) *P* = 0.017 Change in Adj *R*^2^ = 0.042
Highest inotrope score^a^	−0.41(−0.86, −0.04) *P* = 0.074					
**5. Before discharge**						
Post-operative ventilation, days	−0.36(−0.70, −0.01) *P* = 0.042					
Post-operative ICU, days	−0.27(−0.55, 0.01) *P* = 0.057					
Sepsis^b^	−12.10(−20.98, −4.53) *P* = 0.008					−9.70(−17.74, −1.66) *P* = 0.019 Change in Adj *R*^2^ = 0.050
Open sternum, days	−1.33(−2.51, −0.16) *P* = 0.027					
Total explained variance in each time point		13.7%	17.7%	17.7%	26.2%	26.3%

*Coefficient, Effect Size (ES) with 95% confidence intervals (CI), by univariate linear regression (variables with P < 0.10 included) and multiple linear regression at 5 different time points*.

*PB, cardiopulmonary bypass; CPR, cardiopulmonary resuscitation; ECMO, extracorporeal membrane oxygenation; ICU, intensive care unit; ES, effect size; CI, confidence interval*.

a *Inotrope score (22) = dopamine (ug/kg/min) + dobutamine (ug/kg/min) + 100 × epinephrine (ug/kg/min)*.

b *Definition of sepsis (23)*.

## Discussion

This study adds “gestation-related information” to the mortality and developmental outcome information of preterm neonates with CCHD (5–12, 24, 25), and may be useful for the in-hospital management of patients and families including but not limited to (a) the timing of surgical correction, (b) the identification and correction of risk factors that are associated with adverse neurodevelopmental outcomes, (c) parental counseling on the course, care plan, possible outcomes, and importance of follow-up and early interventions, and (d) informing decision-making so clinicians have a better understanding of the course and outcome of these critically-ill preterm neonates.

The preterm neonates of this study with CCHD (mean GA of 34.4 weeks; mean birthweight of 2,339 g), show a high mortality (18.3%) at 2 years of corrected age. When compared with preterm infants of lower GA (31–32 weeks) in the Canadian Neonatal Network, those with CCHD have more neonatal short-term complications including brain injuries (6.1% vs. 1–2% of PVL and parenchymal lesions) and necrotizing enterocolitis (6.1 vs. 1%), but not of retinopathy of prematurity (0 vs. 0% of Stage 3/4/5) (26). The prevalence of cerebral injuries with known association with disability in this group of moderate-late preterm and modestly low birthweight infants was high, 7 (6.1%). Chronic lung disease and patent ductus arteriosus were not examined in this cohort because the respective conditions were difficult to diagnose in surviving neonates with co-morbid cardiopulmonary condition of CCHD after OHS. Growth of these 2-year-old children was within normal ranges, although skewed to the left of population norms.

Consistent with previous reports (13, 27, 28), we have shown the primary cardiac defect of single ventricle and the peri-operative risk factor of CPR are important determinants of mortality. While GA and body weight did not predict death, the intra-uterine growth of the fetus as reflected by low birthweight *z-*scores was critical for this prediction. As part of the consideration of termination of pregnancy for a fetus with CCHD, it is important to examine fetal growth rather than GA alone, in addition to the compounding effect of CCHD. Postnatal age and post-conceptual age of surgery were not related to mortality. This finding should be interpreted with caution because of the small study population. We speculate that postnatal transition of cardiopulmonary systems may affect the tolerance of OHS, a pathophysiological phenomenon which is similar to the delay of surgery in infants with congenital diaphragmatic hernia (29). Therefore, based on the relation between outcome and postnatal and post-conceptual age, we suggest the OHS should be postponed to the time after feto-neonatal transition with the stabilization of cardiopulmonary status.

This study provides information about the early childhood outcomes that may be useful for counseling parents of preterm neonates with CCHD. Dysmaturation and dysregulation of cortical neuronal development due to reduced cerebral oxygenation in CCHD might be responsible for neurodevelopmental adversity (30, 31). Cerebral palsy was found in 10.5% for those without syndromic diagnoses; this suggests higher rates of cerebral palsy than for preterm survivors of the same GA without CCHD (32, 33) and higher than those of mostly term infants with CCHD (34). This study also suggests that sensorineural hearing loss was higher than that for preterm infants without CCHD (35, 36) and similar to term infants with CCHD (37). Vision loss was not increased for these preterm children over other preterm rates (35, 36). This study did not compare CCHD children with and without prematurity, however, mean scores for functional and neurodevelopmental outcomes of preterm neonates with CCHD in this study tend to be within the lower range of published results for preterm neonates (33, 35, 36). Particularly, low scores for self-care skills and language abilities indicate the need for early developmental testing and appropriate interventions for these children (38–40).

Among the predictive factors, the presence of syndromic diagnoses adversely affected neurodevelopmental outcome, as previously reported (41). Within birthweight subgroups, a birthweight of 2,000–2,499 g had a negative (unexpected) correlation with ABAS-II GAC in 76 preterm neonates without syndromic abnormalities. While Dimmick et al. (42) observed that low birthweight was associated with adverse outcomes in infants with CCHD, we do not know the exact reason to explain this relationship, which warrants replication to determine its importance. Due to the retrospective study design and small sample size, risk factors including CPR, inotrope scores, GA, birthweight *z-*scores and postnatal age of surgery had modest or no effects on the ABAS-II-GAC. Older post-conceptual age, low post-operative pH and sepsis added to the prediction. We have previously shown the significant effect of sepsis on adverse outcomes after early cardiac surgery (23). Using multiple variable analyses for prediction of ABAS-II GAC at 2 years corrected age, the cumulative variance explained was 26.3% in preterm infants without syndromic diagnoses. This highlights the possible importance of post-discharge risk factors in subsequent neurodevelopment, in addition to possible risk factors during pregnancy and hospitalization that we did not study.

Our study has several limitations. The research objective was retrospectively determined. Single center study and changes in clinical practice and surgical interventions over a span of 25 years, some antenatal risk factors were missed. This includes but limits to the study of cardiovascular and cerebrovascular state. Small sample size limited us from finding statistically significant relationships. In particular, there were only 6 infants with brain injuries identified in the pre-operative period and thus we could not study its predictive role in neurodevelopment in the multiple variable model. However, information on brain injury before surgery would be helpful in counseling. We did not have detailed information of the maternal conditions associated with the preterm births. Future investigations should address if these conditions may have impact on *in utero* growth or adverse post-natal effects on the infants which are independent of the gestational age. Given these limitations, our findings should be interpreted with caution and generalization to other centers. The use in parental counseling may be limited. Further multicenter prospective study will be needed to confirm our findings. Strengths of this study include the prospective inception cohort design with most data collected prospectively, and the detailed neurodevelopmental and functional outcomes determined prospectively without any loss to follow-up of survivors. Of note, the infants who did not survive were sicker than the survivors. While it may be interesting to study the prediction of a combined group of adverse outcomes including non-survivors and disabled survivors, the current study design was to study the prediction of mortality and neurodevelopmental outcomes in a separate manner.

## Conclusions

Preterm neonates with CCHD and early OHS had significant mortality and morbidity. Early outcomes suggest more cerebral palsy and lower functional and neurodevelopmental scores than occurring with prematurity alone. In this preterm group, predictive factors for mortality included birthweight *z-*scores, cardiac lesion of single ventricle, prolonged post-operative ventilation and the need for CPR. In addition to syndromic diagnoses, predictive factors for adverse functional outcome (ABAS-II GAC) included birth weight 2,000–2,499 g, older post-conceptual age of surgery, post-operative acidosis, and sepsis. These findings may aid clinicians in discussing outcomes with families. While parents are anxious and stressed regarding the prognosis of these infants with CCHD, it may be difficult to explain to the parents and counsel them regarding the impact of birth weight category and post-conceptual age on the outcomes. Nevertheless, the information may help explain the management of these infants including the timing of delivery and surgery. Future studies may assist in providing better counseling. Furthermore, these findings emphasize the importance of long-term follow-up of survivors to detect neurodevelopmental concerns that can be addressed by early intervention programs, ensuring these vulnerable children can reach their full potential.

## Data Availability Statement

The raw data supporting the conclusions of this article will be made available by the authors, without undue reservation.

## Ethics Statement

The studies involving human participants were reviewed and approved by Institutional ethics boards at all six follow-up sites (University of British Columbia, University of Alberta, University of Calgary, University of Regina, University of Saskatchewan, and University of Manitoba). Written informed consent to participate in this study was provided by the participants' legal guardian/next of kin.

## Author Contributions

P-YC conceptualized and designed the study, drafted the initial manuscript, reviewed, and revised the manuscript. CR designed the data collection instruments, conceptualized and designed the study, supervised data collection, critically reviewed, and revised the manuscript. MH and ID carried out the statistical analyses, and critically reviewed and revised the manuscript. HS and AJ conceptualized and designed the study, supervised data collection, and critically reviewed the manuscript for important intellectual content. GB conceptualized and designed the study, coordinated data collection, and critically reviewed the manuscript for important intellectual content. All authors approved the final manuscript as submitted and agree to be accountable for all aspects of the work.

## Conflict of Interest

The authors declare that the research was conducted in the absence of any commercial or financial relationships that could be construed as a potential conflict of interest.

## Abbreviations

ABAS-II, Adaptive Behavior Assessment System: second edition
CCHD: complex congenital heart defects
CI: confidence interval
CPB: cardiopulmonary bypass
CPR: cardiopulmonary resuscitation
ES: effect size
GA: gestational age
GAC: General Adaptive Composite
IQR: interquartile range
OHS: open heart surgery
OR: odds ratio
SD: standard deviation.

## References

[1] O'Brien SM Clarke DR Jacobs JP Jacobs ML Lacour-Gayet FG Pizarro C. An empirically based tool for analyzing mortality associated with congenital heart surgery. J Thorac Cardiovasc Surg. (2009) 138:1139–53. 10.1016/j.jtcvs.2009.03.071 19837218

[2] Gaynor JW Wernovsky G Jarvik GP Bernbaum J Gerdes M Zackai E. Patient characteristics are important determinants of neurodevelopmental outcomes at one year of age after neonatal and infant cardiac surgery. J Thorac Cardiovasc Surg. (2007) 133:1344–53. 10.1016/j.jtcvs.2006.10.087 17467455 PMC2844117

[3] Behrman RE Stith Butler A Institute of Medicine Committee on Understanding Premature Birth and Assuring Healthy Outcomes Board on Health Sciences Outcomes: Preterm Birth: Causes, Consequences, and Prevention. Washington, DC: The National Academies Press (2007). 20669423

[4] Raju TN Higgins RD Stark AR Leveno KJ Optimizing care and outcome for late-preterm (near-term) infants: a summary of the workshop sponsored by the National Institute of Child Health and Human Development. Pediatrics. (2006) 118:1207–14. 10.1542/peds.2006-0018 16951017

[5] Chang AC Hanley FL Lock JE Castaneda AR Wessel DL. Management and outcome of low birth weight neonates with congenital heart disease. J Pediatr. (1994) 124:461–6. 10.1016/S0022-3476(94)70376-0 8120722

[6] Dees E Lin H Cotton RB Graham TP Dodd DA. Outcome of preterm infants with congenital heart disease. J Pediatr. (2000) 137:653–9. 10.1067/mpd.2000.108568 11060531

[7] Costello JM Pasquali SK Jacobs JP He X Hill KD Cooper DS. Gestational age at birth and outcomes after neonatal cardiac surgery: an analysis of the Society of Thoracic Surgeons Congenital Heart Surgery Database. Circulation. (2014) 129:2511–7. 10.1161/CIRCULATIONAHA.113.005864 24795388 PMC4276251

[8] Goff DA Luan X Gerdes M Bernbaum J D'Agostino JA Rychik J Younger gestational age is associated with worse neurodevelopmental outcomes after cardiac surgery in infancy. J Thorac Cardiovasc Surg. (2012) 143:535–42. 10.1016/j.jtcvs.2011.11.029 22340027 PMC4423823

[9] Costello JM Polito A Brown DW McElrath TF Graham DA Thiagarajan RR. Birth before 39 weeks' gestation is associated with worse outcomes in neonates with heart disease. Pediatrics. (2010) 126:277–84. 10.1542/peds.2009-3640 20603261

[10] Cnota JF Gupta R Michelfelder EC Ittenbach RF. Congenital heart disease infant death rates decrease as gestational age advances from 34 to 40 weeks. J Pediatr. (2011) 159:761–5. 10.1016/j.jpeds.2011.04.020 21676411

[11] Curzon CL Milford-Beland S Li JS O'Brien SM Jacobs JP Jacobs ML. Cardiac surgery in infants with low birth weight is associated with increased mortality: analysis of the Society of Thoracic Surgeons Congenital Heart Database. J Thorac Cardiovasc Surg. (2008) 135:546–51. 10.1016/j.jtcvs.2007.09.068 18329467

[12] Hickey EJ Nosikova Y Zhang H Caldarone CA Benson L Redington A. Very low-birth-weight infants with congenital cardiac lesions: is there merit in delaying intervention to permit growth and maturation? J Thorac Cardiovasc Surg. (2012) 143:126–36. 10.1016/j.jtcvs.2011.09.008 22018991

[13] Robertson CM Joffe AR Sauve RS Rebeyka IM Phillipos EZ Dyck JD. Outcomes from an interprovincial program of newborn open heart surgery. J Pediatr. (2004) 144:86–92. 10.1016/j.jpeds.2003.09.048 14722524

[14] Cheung PY Chui N Joffe AR Rebeyka IM Robertson CM; Western Canadian Complex Pediatric Therapies Project Follow-up Group Postoperative lactate concentrations predict the outcome of infants aged 6 weeks or less after intracardiac surgery: a cohort follow-up to 18 months. J Thorac Cardiovasc Surg. (2005) 130:837–43. 10.1016/j.jtcvs.2005.04.029 16153937

[15] Centers for Disease Control and Prevention National Center for health Statistics CDC Growth Charts, United States. (2009) Available online at: http://www.cdc.gov/growth~charts/ (accessed April, 2020).

[16] Fenton TR Kim JH PediTools: clinical tools for pediatric providers, based on the CDC growth calculator for 0–36 months for percentiles and *Z*-scores. BMC Pediatrics. (2013) 13:59 10.1186/1471-2431-13-59 23601190 PMC3637477

[17] Harrison PL Oakland T Manual of the Adaptive Behaviour Assessment System II. Psychological Corp, Harcourt Assessment Company (2003).

[18] Bayley N Bayley Scales of Infant and Toddler Development. 3rd ed San Antonio, TX: Harcourt Assessment Inc (2006).

[19] Rosenbaum P Paneth N Leviton A Goldstein M Bax M A report: the definition and classification of cerebral palsy. Dev Med Child Neurol. (2007) 49(Suppl.109):8–14. 17370477

[20] Blishen B Carroll W Moore C The 1981 socioeconomic index for occupations in Canada. Can Rev Social Anthropol. (1987) 24:465–88. 10.1111/j.1755-618X.1987.tb00639.x

[21] R Core Team R: A Language and Environment for Statistical Computing. Vienna, Austria: R Foundation for Statistical Computing (2019). Available online at: https://www.R-project.org/ (accessed April, 2020)

[22] Wernovsky G Wypij D Jonas RA Mayer JE Jr Hanley FL Hickey PR. Post-operative course and hemodynamic profile after the arterial switch operation in neonates and infants. A comparison of low-flow cardiopulmonary bypass and circulatory arrest. Circulation. (1995) 92:2226–35. 10.1161/01.CIR.92.8.2226 7554206

[23] Sidhu N Joffe AR Doughty P Vatanpour S Dinu I Alton G. Sepsis after cardiac surgery early in infancy and adverse 4.5-year neurodevelopmental outcomes. J Am Heart Assoc. (2015) 4:e001954. 10.1161/JAHA.115.001954 26251282 PMC4599458

[24] Chu PY Li JS Kosinski AS Hornik CP Hill KD. Congenital heart disease in premature infants 25-32 weeks' gestational age. J Pediatr. (2017) 181:37–41. 10.1016/j.jpeds.2016.10.033 27816222 PMC5274591

[25] Lytzen R Vejlstrup N Bjerre J Petersen OB Leenskjold S Dodd JK. Live-born major congenital heart disease in Denmark: Incidence, detection rate, and termination of pregnancy rate from 1996 to 2013. JAMA Cardiol. (2018) 3:829–37. 10.1001/jamacardio.2018.2009 30027209 PMC6233653

[26] Canadian Neonatal Network™ Annual Report 2018 (2019). Available online at: http://www.canadianneonatalnetwork.org/Portal/LinkClick.aspx?fileticket=PvniYH94zm0%3d&tabid=39 (accessed April, 2020)

[27] Alsoufi B Slesnick T McCracken C Ehrlich A Kanter K Schlosser B. Current outcomes of the Norwood operation in patients with single-ventricle malformations other than hypoplastic left heart syndrome. World J Pediatr Congenit Heart Surg. (2015) 6:46–52. 10.1177/2150135114558069 25548343

[28] Kuraim GA Garros D Ryerson L Moradi F Dinu IA Guerra GG. Predictors and outcomes of early post-operative veno-arterial extracorporeal membrane oxygenation following infant cardiac surgery. J Intensive Care. (2018) 6:56. 10.1186/s40560-018-0326-4 30202528 PMC6122608

[29] Puligandla PS Skarsgard ED. The Canadian Pediatric Surgery Network congenital diaphragmatic hernia evidence review project: developing national guidelines for care. Paediatr Child Health. (2016) 21:183–6. 10.1093/pch/21.4.183 27429569 PMC4934157

[30] Leonetti C Back SA Gallo V Ishibashi N. Cortical dysmaturation in congenital heart disease. Trends Neurosci. (2019) 42:192–204. 10.1016/j.tins.2018.12.003 30616953 PMC6397700

[31] Clouchoux C du Plessis AJ Bouyssi-Kobar M Tworetzky W McElhinney DB Brown DW. Delayed cortical development in fetuses with complex congenital heart disease. Cereb Cortex. (2013) 23:2932–43. 10.1093/cercor/bhs281 22977063

[32] Robertson CMT Watt MJ Yasui Y. Changes in the prevalence of cerebral palsy for children born very prematurely within a population-based program over 30 years. JAMA. (2007) 297:2733–40. 10.1001/jama.297.24.2733 17595274

[33] Allotey J Zamora J Cheong-See F Kalidindi M Arroyo-Manzano D Asztalos E. Cognitive, motor, behavioural and academic performances of children born preterm: a meta-analysis and systematic review involving 64061 children. Br J Obstet Gynaecol. (2018) 125:16–25. 10.1111/1471-0528.14832 29024294

[34] Ricci MF Andersen JC Joffe AR Watt MJ Moez EK Dinu IA. Chronic neuromotor disability after complex cardiac surgery in early life. Pediatrics. (2015) 36:e922–33. 10.1542/peds.2015-1879 26391946

[35] Radic JA Vincer M McNeely PD Outcomes of intraventricular hemorrhage and posthemorrhagic hydrocephalus in a population-based cohort of very preterm infants born to residents of Nova Scotia from 1993 to 2010. J Neurosurg Pediatr. (2015) 15:580–8. 10.3171/2014.11.PEDS14364 26030329

[36] Synnes A Luu TM Moddemann D Church P Lee D Vincer M. Determinants of developmental outcomes in a very preterm Canadian cohort. Arch Dis Child Fetal Neonatal Ed. (2017) 102:F235–4. 10.1136/archdischild-2016-311228 27758929

[37] Bork KT To BP Leonard NJ Douglas CM Dinon DA Leonard EE. Prevalence of childhood permanent hearing loss after early complex cardiac surgery. J Pediatr. (2018) 198:104–9. 10.1016/j.jpeds.2018.02.037 29631768

[38] Alton GY Taghados S Joffe AR Robertson CM Dinu I Western Canadian Pediatric Therapies Follow-Up Group. Prediction of preschool functional abilities after early complex cardiac surgery. Cardiol Young. (2015) 25:655–62. 10.1017/S1047951114000535 24784451

[39] Martin BJ De Villiers Jonker I Joffe AR Bond GY Acton BV Hypoplastic left heart syndrome is not associated with worse clinical or neurodevelopmental outcomes than other cardiac pathologies after the Norwood-Sano operation. Pediatr Cardiol. (2017) 38:922–31. 10.1007/s00246-017-1598-5 28341901

[40] Ricci MF Martin BJ Joffe AR Dinu IA Alton GY Guerra GG. Deterioration of functional abilities in children surviving the Fontan operation. Cardiol Young. (2018) 28:868–75. 10.1017/S1047951118000537 29690942

[41] Gaynor JW Stopp C Wypij D Andropoulos DB Atallah J Atz AM. Neurodevelopmental outcomes after cardiac surgery in infancy. Pediatrics. (2015) 135:816–25. 10.1542/peds.2014-3825 25917996 PMC4533222

[42] Dimmick S Walker K Badawi N Halliday R Cooper SG Nicholson IA. Outcomes following surgery for congenital heart disease in low-birthweight infants. J Paediatr Child Health. (2007) 43:370–5. 10.1111/j.1440-1754.2007.01082.x 17489827

